# Excision of Spina Bifida Tumor—Cure

**Published:** 1880-04

**Authors:** W. H. Fitch

**Affiliations:** Rockford, Ill.


### NOTES FROM PRIVATE PRACTICE.

### ARTICLE VII.

*Excision of Spina Bifida Tumor—Cure.*

Although this operation has apparently in this case, resulted in success, I do not report the case thinking any one will imitate my practice, but that the mistake may be avoided in similar cases.

Mrs. D., from the country, brought her child one year old to me, Nov. 24th, 1879, to get advice concerning a tumor on the child's back, in the lumbar region of spine; said they had not noticed the tumor until some time after birth; that it had grown with the growth of the child; that it gave it no inconvenience, except that pressure occasionally caused the child to cry; that it was not accompanied by paralysis of any organ or muscle.

Upon inspection, I found a tumor over lumbar spine about ($2\frac{1}{2}$ in.) six cm. long transversely, four cm. vertically and projecting ($1\frac{1}{2}$ in.) four cm. above surrounding surface and pointing somewhat upward along the spine spreading out at its base into dense, apparently infiltrated tissue. Quite a number of hairs from ($\frac{1}{2}$ in. to $1\frac{1}{2}$ in.) one to three cm. long grew on the tumor near its junction with the back. The tumor was inelastic, non-fluctuating, incompressible in any position of the child, crying or quiet. The absence of any signs of spina bifida except location, led to its removal. Its increased size began to be a source of annoyance and the reddish appearance of the apex of the tumor caused me to fear ulceration. I imagined it to be some species of sarcoma. As the case seemed a little anomalous, I invited three other physicians to examine the case at the time of operation, all of whom concurred in the advisability of its removal. Accordingly on Nov. 25th, the child was chloroformed and an incision was made around the tumor nearly ($\frac{7}{8}$ in.) two cms. from its union with the back, to remove as much as possible of infiltrated tissue in which the tumor seemed deposited. The sensation conveyed to the knife, was as if cutting fibrous dense tissue and not adipose with which the child was abundantly supplied. From the bottom of the wound there welled up a stream of limpid fluid, and it was at once manifest that I had opened a hernia of spinal membranes; in other words, I had to deal with a spina bifida. A ligature was put around the duct, the wound brought together by sutures, compress and bandage applied.

Nov. 26.—Found the bandages and compresses thoroughly wet, as well as several other cloths that had been applied. It was evident that the ligature had come away, and that spinal fluid was being lost in considerable quantities. The child had slept well and took nourishment as usual.

Nov. 29.—From the number of pads, compresses and cloths saturated with this now clear fluid, I judged the amount escaping must have been three pints, although the ladies in attendance assert double the amount. I have since been sorry that the quantity was not collected, as it seemed enormous. The child has lost flesh during last four days and grown pale; appetite fitful, pulse 130, temperature normal; starts some in its sleep; sutures removed, union seemed complete except at one angle, where fluid was discharging. Rubber-coated adhesive plaster was applied and compresses. Ordered (ʒiss) six grams cod-liver oil and (ʒi) four grams elixir calasayæ and iron, three times a day.

Nov. 30.—Found adhesive straps off and wound opened to about original size.

Dec. 2.—Have tried various kinds of adhesive plaster, but some constituents of the fluid detaches them, whether applied by heat or moisture. Ordered that the wound be dressed with carbolated oil. It has shown no disposition to heal although the amount of fluid for last twenty-four hours has considerably diminished. Child looks very feeble.

Dec. 5.—The duct probably closed yesterday, as the flow ceased and wound is contracting rapidly. Child looks somewhat brighter.

Dec 10.—Wound pretty much cicatrized and child regaining flesh and strength as rapidly as it lost it; returns home to-morrow.

The base of the tumor was cavernous, i. e., the canal leading from the spine was dilated into a cavity as large as a hazel-nut, from which there were prolongations in various directions, but none of these extended upward into the tumor proper, or into that part of it projecting above the surface of back. It is now three months since the operation, and the child is well.

ROCKFORD, ILL. W. H. FITCH, M.D.

